# Physics-informed hybrid GR4J–XGBoost model for streamflow prediction: integrating conceptual states, SHAP interpretability, and uncertainty analysis

**DOI:** 10.1038/s41598-026-64700-8

**Published:** 2026-08-03

**Authors:** Ozgur Kisi, Salim Heddam, Kulwinder Singh Parmar, Christoph Külls, Mohammad Zounemat-Kermani

**Affiliations:** 1 Department of Civil Engineering, Technische Hochschule Lübeck, 23562 Lübeck, Germany; 2 Department of Civil Engineering, Ilia State University, 0162 Tbilisi, Georgia; 3 School of Civil, Environmental and Architectural Engineering, Korea University, Seoul, 02841 South Korea; 4 Hydraulics Division, Faculty of Science, Agronomy Department, University 20 Août 1955 Skikda, Route El Hadaik, BP 26, Skikda, Algeria; 5 Department of Mathematics, IKG Punjab Technical University, Jalandhar, Kapurthala, India; 6 Department of Civil Engineering, Shahid Bahonar University of Kerman, Kerman, Iran

**Keywords:** Rainfall–runoff modeling, Hybrid modeling, GR4J model, XGBoost, Physics-informed machine learning, SHAP analysis, Model interpretability, Prediction reliability, Climate sciences, Environmental sciences, Hydrology, Mathematics and computing

## Abstract

Accurate streamflow prediction remains challenging due to the nonlinear and dynamic nature of rainfall–runoff processes. Conceptual hydrological models provide physically interpretable representations, yet their predictive performance is often limited, while data-driven models may suffer from overfitting and a lack of interpretability. To address these limitations, this study proposes a physics-informed hybrid modeling framework that integrates the GR4J conceptual model with the XGBoost machine learning algorithm. Unlike conventional hybrid approaches, the proposed method incorporates GR4J-derived state and flux variables (including simulated discharge, storage state, effective rainfall, evapotranspiration, percolation, and exchange fluxes) as input features, enabling a process-based representation of catchment dynamics within the machine learning framework. The proposed framework was evaluated using a catchment selected from the CAMELS-DE (Catchment Attributes and Meteorology for Large-sample Studies–Germany) dataset. Model performance was assessed using multiple lag configurations (7, 14, and 30 days) across training, validation, and independent test datasets. The results demonstrate that the hybrid GR4J–XGBoost model generally outperforms the standalone GR4J and XGBoost models, particularly in terms of generalization capability and predictive stability. While the standalone XGBoost model benefits from longer lag structures, the hybrid model achieves comparable or superior predictive performance with shorter lag inputs, indicating reduced dependence on extended temporal memory while providing physically meaningful conceptual information for model interpretation. SHAP analysis reveals that physically meaningful variables, especially GR4J-derived state and flux components, play a dominant role in governing model predictions. Furthermore, residual-based uncertainty assessment shows that the hybrid model produces lower prediction bias, reduced residual dispersion, and narrower residual uncertainty bounds than the standalone approaches, indicating improved prediction reliability. Overall, the proposed framework provides a physics-informed, process-based, and interpretable approach for rainfall–runoff modeling while offering an additional assessment of prediction reliability through residual-based uncertainty evaluation.

## Introduction

The multifaceted interactions between climate forcing, catchment storage, and drainage processes involve intricate nonlinear dynamics in specific hydrological events such as rainfall-runoff responses^1,2^. In this regard, this complexity is compounded by highly variable hydro-climatic conditions, diverse watershed geomorphology, and the influence of both natural phenomena and human-made structures. One main reason for this complexity is the scale-dependent nature of the hydrological responses to rainfall-runoff phenomena, where processes operating at small scales (such as soil moisture infiltration, evapotranspiration, and surface storage) aggregate nonlinearly to produce river flow patterns at the catchment scale. Given the lack of complete access to information at this scale, accurate and reliable modeling of this process is difficult in practice, and in some cases involves significant error^3, 4, 5^.

Over the past years, many researchers have tried to create and develop conceptual, mathematical, and data-driven hydrological models^6^. Among these, conceptual rainfall-runoff models have been widely used due to their relatively low computational cost and interpretable physical state variables. Examples include the widely recognized GR4J model^7^, TOPMODEL (Holko and Lepistö^8^), the SAC-SMA model^9^, the HEC-HMS model^10^, as well as other significant models like HBV^11^, and the Soil & Water Assessment Tool (SWAT)^12^. These models simulate hydrological processes by taking meteorological and morphological information from the catchment, performing calculations related to soil moisture and flow routing, and then providing aggregate or semi-distributed time series of flow components, giving insight into how water moves within the watershed system and about the overall system response.

Conversely, data-driven types of Machine Learning (ML) models have shown that they can produce significant and powerful predictions for complex hydrological datasets and their relationships^13^. Of all these types of data-driven models, the most recent ensemble ML models, such as gradient boosting models, like XGBoost, have gained significant popularity for hydrological predictions due to their high efficiency, resistance to overfitting, and ability to efficiently accommodate complex relationships^14^. Indeed, some recent studies showed that boosting methodologies can perform equally efficiently for streamflow predictions as deep learning models, with greater ability for interpretation using the feature importance approach^15,16^. However, one of the major shortcomings of the ML models is their inability to provide a transparent interpretation of the obtained simulated results. ML models are often considered black-box models, which means their internal architecture is opaque and typically does not provide physical interpretability. As such, many users perceive issues of consistency and robustness beyond the training domain^17^. These predictions provide accurate results but will not necessarily capture the underlying hydrological processes, making them unreliable for scenarios that are different from those presented in the training data.

Recent research has increasingly adopted hybrid modelling approaches that combine machine learning (ML) components with conceptual hydrological models to preserve physical consistency while improving predictive performance^18–20^. In terms of interpretability, these hybrid models may be categorized as in-mode explainable artificial intelligence (XAI) methods since they embed physical constraints and domain knowledge directly into the learning process, ensuring that explanatory mechanisms are active throughout model development rather than being applied after training or designed solely before it^21^. There are several possible methods of integration, including but not limited to multi-model data fusion^22^, physics-guided machine learning where physical constraints are embedded into loss functions^23^, and feature augmentation using conceptual model outputs as additional machine learning inputs^24^. Humphrey et al.^19^ reported that integrating a conceptual model with artificial neural networks produced improved monthly flow predictions, and several hybrid models, such as GR4J-LSTM have been shown to enhance daily simulation of streamflow^25^. More recently, Kapoor et al.^26^ proposed the DeepGR4J framework, which combines the production-storage component of GR4J with deep learning architectures (CNN and LSTM) to improve rainfall–runoff simulation. While this framework demonstrated the potential of conceptual feature augmentation for improving predictive performance, it primarily relied on deep neural networks and did not investigate explainable machine learning or predictive uncertainty within the hybrid framework. More recently, additional studies have advanced runoff prediction through increasingly sophisticated deep learning architectures that integrate feature decomposition, attention mechanisms, transformer-based learning, and optimization strategies^27,28^. These studies have demonstrated remarkable improvements in predictive accuracy. However, their primary emphasis remains on enhancing network architectures and feature learning, while limited attention has been given to incorporating physically meaningful conceptual hydrological variables, explainable machine learning, and uncertainty quantification within a unified modelling framework. Despite these advances, several important research gaps remain. Existing GR4J-based hybrid models have largely focused on deep neural network architectures, whereas comparatively little attention has been given to gradient-boosting methods capable of providing competitive predictive performance with enhanced interpretability. In addition, the individual contribution of conceptual hydrological states has rarely been systematically quantified using explainable AI techniques, and residual-based uncertainty assessment has seldom been incorporated into hybrid modelling frameworks to evaluate prediction reliability.

In addition to physics-guided ML models, other recent alternative techniques for interpreting the outcomes of ML black-box models are post-hoc XAI. Notable model-agnostic methods include SHAP (SHapley Additive exPlanations), LIME (Local Interpretable Model-agnostic Explanations), Partial Dependence Plots (PDP), and Accumulated Local Effects (ALE) plots^29,30^. They show which factors have the strongest effect on a prediction and increase trust in the result. In this way, the interaction between the physical water component plus the data-driven component inside a combined model becomes visible. Instead of speculating, researchers now measure how far each input alters the output and obtain firm evidence of its influence. SHAP gives a reliable way to see how soil moisture, plant evapotranspiration, or estimated river discharge steer the model. With this connection, forecast accuracy and insight into water movement are both achieved together^31^. Nonetheless, while SHAP and related methods can reveal feature importance in hybrid or black-box models, they do not necessarily ensure that learned relationships by the applied ML models are hydrologically consistent or even generalize beyond training conditions.

Despite these promising developments, several important research gaps remain in developing hybrid hydrological models that are simultaneously physics-informed, interpretable, and capable of providing uncertainty estimates. However, the contribution of intermediate conceptual variables to hybrid model behaviour has received comparatively little attention and has rarely been systematically evaluated using ablation analysis. Although post-hoc XAI methods such as SHAP can explain model predictions after training, they cannot guarantee that the learning process itself is guided by hydrologically meaningful information. Unlike previous GR4J-based hybrid frameworks that primarily emphasize predictive accuracy using deep neural networks, the proposed approach integrates physically meaningful conceptual state variables with a gradient-boosting model, explainable AI, and residual-based uncertainty analysis within a unified physics-informed framework. To address these limitations, this study proposes a physics-informed GR4J–XGBoost hybrid framework that combines conceptual hydrological state variables, explainable artificial intelligence, and residual-based uncertainty assessment within a unified modelling framework. his integrated approach is intended to maintain competitive predictive performance while enhancing physical interpretability and facilitating the evaluation of prediction reliability. The aim of this study is therefore to create and deploy hybrid hydrological models that are interpretable at the same development stage, not just after the event. Rather than pursuing a broad regional comparison, this study intentionally adopts a detailed single-catchment framework to investigate the interaction between conceptual hydrological information and machine learning under well-controlled hydro-climatic conditions. This design allows the proposed hybrid framework to be evaluated in depth before future extension to multiple catchments. The river basin selected from the CAMELS-DE dataset has extensive and continuous climate records—and clearly defined physical land features—so it is useful for examining the connection between the way we generally understand how water systems work and learning from the data itself. In addition, the proposed framework enables the contribution of intermediate conceptual variables to be systematically investigated through complementary ablation analysis and SHAP-based explainable AI.

To address these research gaps, this study pursues the following objectives:

1. Integration of GR4J state variables (e.g., net evapotranspiration demand, production store inflow, actual evaporation, percolation, production store storage, and simulated discharge) into a gradient-boosting framework to leverage physically meaningful hydrological information.
2. Systematic evaluation of temporal lag effects (7, 14, and 30 days) on rainfall–runoff prediction skill to identify optimal memory lengths for the hybrid model.
3. Evaluation of prediction reliability through residual-based uncertainty assessment using statistical error measures (ME, SD, lower and upper uncertainty bounds, and WUCB), thereby complementing deterministic performance metrics.
4. Interpretation of hybrid model behaviour through SHAP-based explainable machine learning analysis and evaluation of the contribution of conceptual variables using ablation analysis.
5. Application of the proposed framework to a well-documented catchment from the CAMELS-DE dataset, ensuring transparency and reproducibility of the methodology.

The remainder of this paper is organized as follows. Section “Materials and methods” describes the study area and data. Section “Results” presents the hybrid modeling framework and the residual-based uncertainty assessment methodology. Section “Discussion” discusses the results, followed by conclusions and perspectives for future research in Sect. “Conclusions”.

## Materials and methods

### Case study

**Fig. 1:**
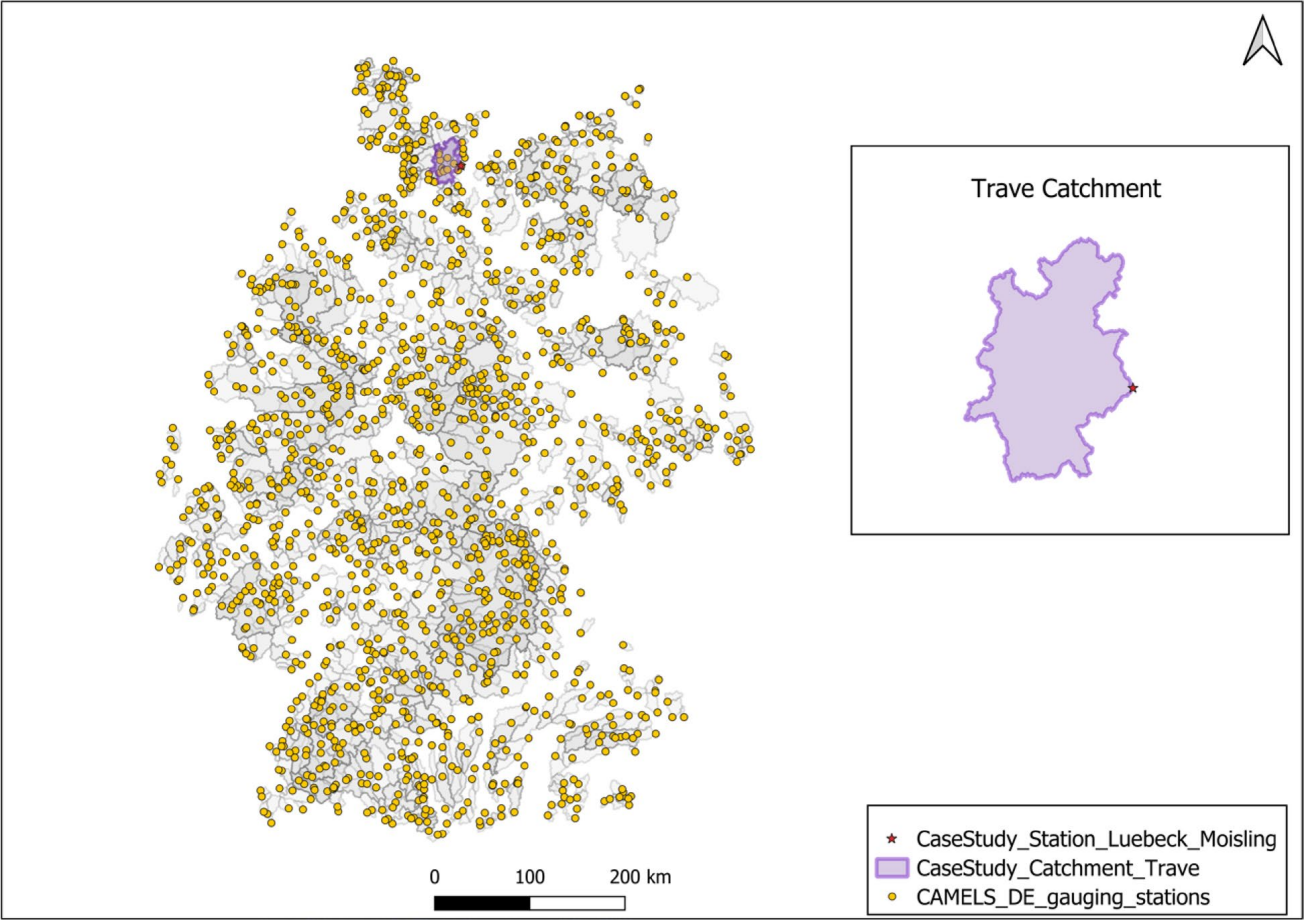
Spatial distribution of CAMELS-DE gauging stations across Germany, along with the location of the selected case study. The Trave catchment and the Lübeck-Moisling gauging station are highlighted. The inset map provides a detailed view of the case study basin.

The study area is located in northern Germany and corresponds to the Trave catchment, with streamflow observations obtained from the Lübeck-Moisling gauging station (Fig. 1). The catchment is part of the CAMELS-DE dataset and is characterized by a temperate climate with moderate precipitation variability and distinct seasonal patterns. The Trave catchment has a drainage area of approximately 912.49 km^2^, providing a representative medium-sized basin for rainfall–runoff analysis. The elevation of the basin ranges from approximately − 4.53 m to 115.07 m, with a mean elevation of 40.44 m, reflecting relatively low-relief topography typical of northern Germany.

Daily time series of streamflow (Q), precipitation (P), and potential evapotranspiration (E) were used as input variables. The dataset was obtained from the CAMELS-DE database, which provides consistent and quality-controlled hydrometeorological observations across Germany. The dataset was preprocessed prior to analysis. A continuous daily dataset covering the period 2011–2020 was selected due to data availability. The dataset was chronologically divided into 60% training, 20% validation, and 20% testing periods to preserve temporal dependency in the rainfall–runoff process. The training, validation, and testing periods approximately correspond to 2011–2016, 2017–2018, and 2019–2020, respectively. The selected catchment exhibits variability in flow conditions, including both low-flow and high-flow events, making it suitable for evaluating the robustness and reliability of hybrid modeling approaches.

### GR4J hydrological model

The GR4J hydrological model was adopted to represent rainfall-runoff processes of the catchment and to provide a physically based component within the proposed hybrid modeling framework. Conceptual hydrological models simplify complex watershed behavior into a set of interconnected storage components that transform meteorological inputs such as precipitation and evapotranspiration into streamflow^7,32^, (Sezen and Partal^33^). Among such models, the GR4J is widely used due to its parsimonious structure, strong physical interpretability, and relatively low number of parameters, and ability to generate state variables that are useful for subsequent data-driven models (Sezen and Partal^33^).

**Fig. 2:**
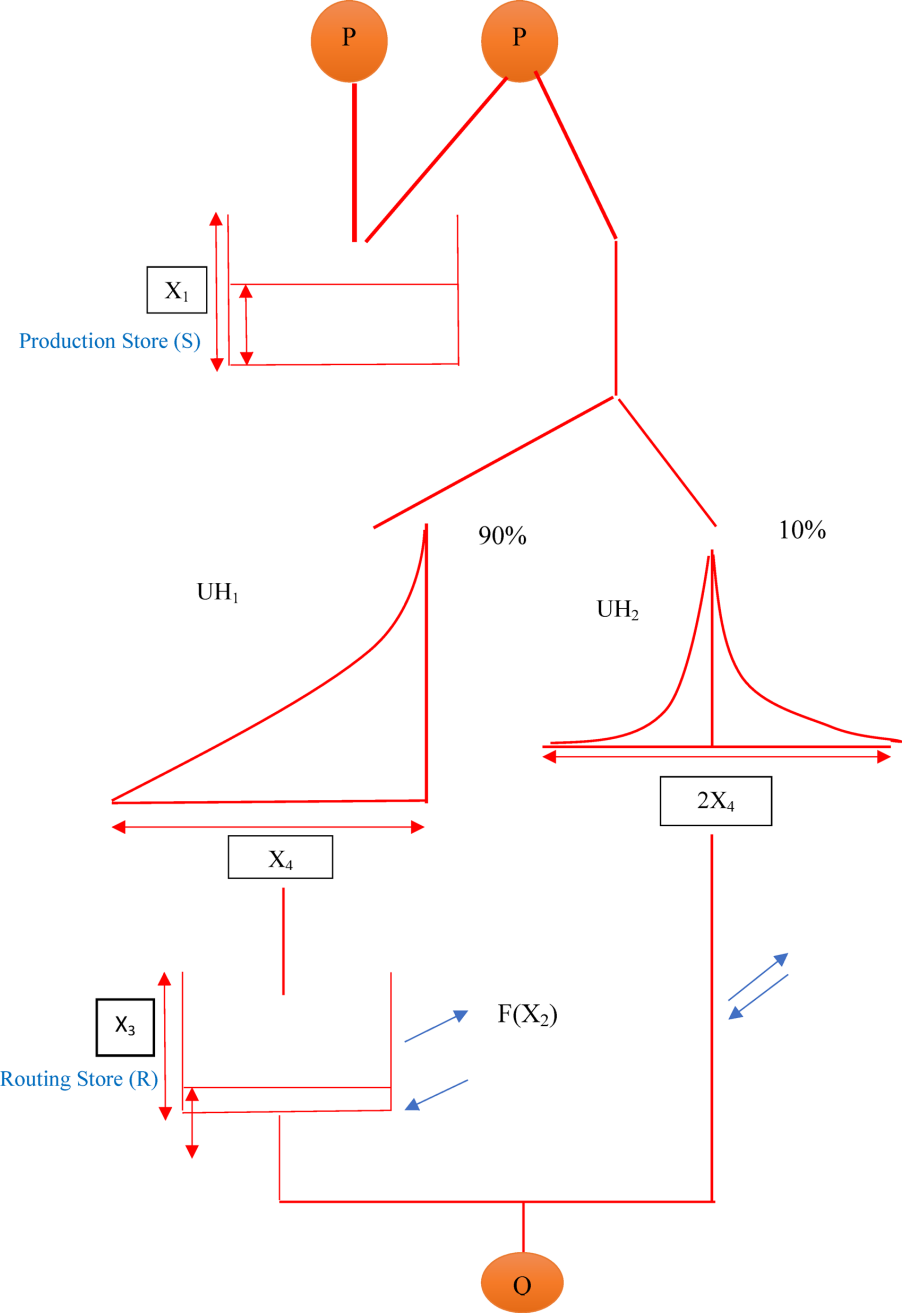
Conceptual framework of the GR4J model.

The GR4J model’s conceptual framework consists of two nonlinear reservoirs—the production store, which stimulates the soil moisture budget associated with the precipitation (P) and evaporation (E) input time series and the routing store, which governs the transformation of effective rainfall into streamflow (Fig. 2). Moreover, these reservoirs are controlled by four parameters, including production store capacity X_1_; groundwater exchange coefficient X_2_; routing store capacity X_3_; and unit hydrograph time base X_4_. The limited number of parameters helps to reduce calibration uncertainty and improves computational efficiency^34^. The parameter (X_1_) governs how much of the water retained by the soil prior to contributing significantly to streamflow. This defines the upper limit of soil retention (in mm) that the production store can hold. When production store is full, the excessive rainfall from this reservoir is split in two flows: a quick flow routed via Unit Hydrograph 1 (UH 1), which represents water reaches the river or outlet very quickly and a slow flow representing the slow response of the catchment and goes into the second reservoir routed via Unit Hydrograph 2 (UH 2). The proportion of the flow going in second water store (called Routing store) is controlled by the parameter (X_2_), which represents ground water storage and delayed flow. As in the first reservoir, maximum capacity in routing store (in mm) is regulated by the parameter (X_3_). In the final phase, time base of unit hydrographs (in days) controls speed of flow to outlet and it corresponds to the concluding variable (X_4_).

At the beginning, net precipitation $\left( {P_{n} } \right)$ is determined by the model by deducting the potential evapotranspiration from the rainfall and same process is followed for computing net evapotranspiration (*E*_*n*_)^26^. If the amount of potential evapotranspiration is greater than the amount of precipitation (*E > P* ), then net precipitation $\left( {P_{n} } \right)$ = 0, and net evapotranspiration becomes $E_{n} = E - P$. On the other hand, if *E łe P*, then net precipitation $P_{n} = P - E$ with *E*_*n*_ = 0^7^. A portion of the net rainfall say $P_{s}$ enters the production reservoir, and the remaining portion $\left( {P_{n} - P_{s} } \right)$ combined with leakage percolation (*Perc*) from the production store/reservoir goes into the flow-routing reservoir. In the production store, the moisture at time t is given by following water balance equation^35^:

1 $$S^{\left( t \right)} = S^{{\left( {t - 1} \right)}} - E_{s}^{\left( t \right)} + P_{s}^{\left( t \right)}$$

where, $P_{s}^{\left( t \right)}$ and $E_{s}^{\left( t \right)}$ are computed as:

2 $$P_{s}^{\left( t \right)} = \frac{{X_{1} \left[ {1 - \left\{ {\frac{{S^{{\left( {t - 1} \right)}} }}{{X_{1} }}} \right\}^{2} } \right]\tan h\left( {\frac{{P_{n}^{\left( t \right)} }}{{X_{1} }}} \right)}}{{1 + \frac{{S^{{\left( {t - 1} \right)}} }}{{X_{1} }}\tan h\left( {\frac{{P_{n}^{\left( t \right)} }}{{X_{1} }}} \right)}}$$

3 $$E_{s}^{\left( t \right)} = \frac{{S^{{\left( {t - 1} \right)}} \left[ {2 - \frac{{S^{{\left( {t - 1} \right)}} }}{{X_{1} }}} \right]\tan h\left( {\frac{{E_{n}^{\left( t \right)} }}{{X_{1} }}} \right)}}{{1 + \left[ {1 - \frac{{S^{{\left( {t - 1} \right)}} }}{{X_{1} }}} \right]\tan h\left( {\frac{{E_{n}^{\left( t \right)} }}{{X_{1} }}} \right)}}$$

The leakage percolation and net precipitation after $P_{s}$ is absorbed by production reservoir are then computed as^26,35^:

4 $$Perc^{\left( t \right)} = S^{\left( t \right)} \left[ {1 - \left\{ {1 + \left( {\frac{4}{9}\frac{{S^{\left( t \right)} }}{{X_{1} }}} \right)^{4} } \right\}^{{\frac{ - 1}{4}}} } \right]$$

5 $$P_{r}^{\left( t \right)} = P_{n}^{\left( t \right)} - P_{s}^{\left( t \right)} + Perc^{\left( t \right)}$$

The combined inflow is divided into two parts: (i) $Q_{9}$ constitutes 90% of the runoff; it is initially processed by a unit hydrograph (UH1) and subsequently directed to the routing reservoir, (ii) The delayed portion of runoff, accounting for 10% of the total, is denoted by $Q_{1}$ and is derived through a single unit hydrograph (UH2). Both these flows are controlled by the parameter X_4_and are mathematically depicted as:

6 $$Q_{9} \left( t \right) = 0.9*\mathop \sum \limits_{k = 1}^{m} HU1\left( k \right) \times P_{r} \left( {t - k + 1} \right)$$

7 $$Q_{1} \left( t \right) = 0.1*\mathop \sum \limits_{k = 1}^{l} HU2\left( k \right) \times P_{r} \left( {t - k + 1} \right)$$

where, the limits *m* and *l* are respectively $X_{4}$ and $2X_{4}$.

After incorporating groundwater interactions $\left( F \right)$ and the outflow from the routing reservoir $\left( {Q_{r} } \right)$, the state of the routing moisture storage (*R*) is updated according to following expression:

8 $$F\left( t \right) = X_{2} \left( {\frac{{R\left( {t - 1} \right)}}{{X_{3} }}} \right)^{\frac{7}{2}}$$

9 $$R\left( t \right) = \max \left( {R\left( {t - 1} \right) + Q_{9} \left( t \right) + F\left( t \right);0} \right)$$

10 $$Q_{r} \left( t \right) = R\left( t \right)\left[ {1 - \left\{ {1 + \left( {\frac{R\left( t \right)}{{X_{3} }}} \right)^{4} } \right\}^{{\frac{ - 1}{4}}} } \right]$$

After computing the direct runoff $Q_{d} \left( t \right)$, the final simulated total stream flow $Q_{sim} \left( t \right)$ is calculated by adding these runoffs together as follows:

11 $$Q_{d} \left( t \right) = \max \left( {0;F\left( t \right){ + }Q_{1} \left( t \right)} \right)$$

12 $$Q_{sim} \left( t \right) = Q_{r} \left( t \right) + Q_{d} \left( t \right)$$

Accurate estimation of the four GR4J parameters (X_1_–X_4_) is critical for reliable streamflow simulation. The four GR4J parameters were calibrated exclusively using the training dataset (60%) through the Levenberg–Marquardt (LM) nonlinear least-squares optimization algorithm implemented in the SciPy optimization library. The LM algorithm combines the advantages of gradient-based optimization with the robustness of the Gauss–Newton method for handling nonlinear least-squares problems, making it well suited for calibrating conceptual hydrological models such as GR4J. The calibration aimed to minimize the error between observed discharge $Q_{obs} \left( t \right)$ and model simulated discharge $Q_{sim} \left( t \right)$. The residual at each time step *t* is defined as

13 $$r\left( t \right) = Q_{obs} \left( t \right) - Q_{sim} \left( t \right)$$

The optimizing objective is mathematically represented as ${\mathit{min}{\sum}_{t=1}^{n}\left(r(t)\right)}^{2}$.

This nonlinear least-squares formulation is mathematically equivalent to maximizing the Nash–Sutcliffe Efficiency (NSE), which is a widely accepted goodness of fit in hydrological modeling and is defined as (Sezen and Partal^33^):

14 $$NSE = 1 - \frac{{\mathop \sum \nolimits_{t = 1}^{n} \left( {r\left( t \right)} \right)^{2} }}{{\mathop \sum \nolimits_{t = 1}^{n} \left( {Q_{obs} \left( t \right) - \overline{{Q_{obs} \left( t \right)}} } \right)^{2} }}.$$

Using the LM optimization algorithm, the calibration converged to the following parameter values: X_1_ = 109.319, X_2_ = 3.196, X_3_ = 192.216, and X_4_ = 1.262. These parameter values represent the final calibrated GR4J model and were subsequently used to simulate the complete streamflow series, from which the conceptual state variables and flux components required by the hybrid XGBoost model were extracted. The same calibrated parameter set was applied to the validation and independent test datasets without further adjustment.

### XGBoost machine learning model

To capture nonlinear relationships between hydrological inputs and streamflow, the Extreme Gradient Boosting (XGBoost) algorithm was employed. XGBoost is an efficient implementation of gradient boosted decision trees that combine high predictive accuracy with regularization to prevent overfitting. This algorithm has gained significant popularity in hydrological modeling and environmental prediction due to its ability to learn nonlinear dynamics from large and heterogeneous datasets while maintaining robustness against overfitting. In this study, XGBoost was selected because of its ability to model complex nonlinear rainfall–runoff relationships while effectively utilizing the physically meaningful variables generated by the GR4J model.

**Fig. 3:**
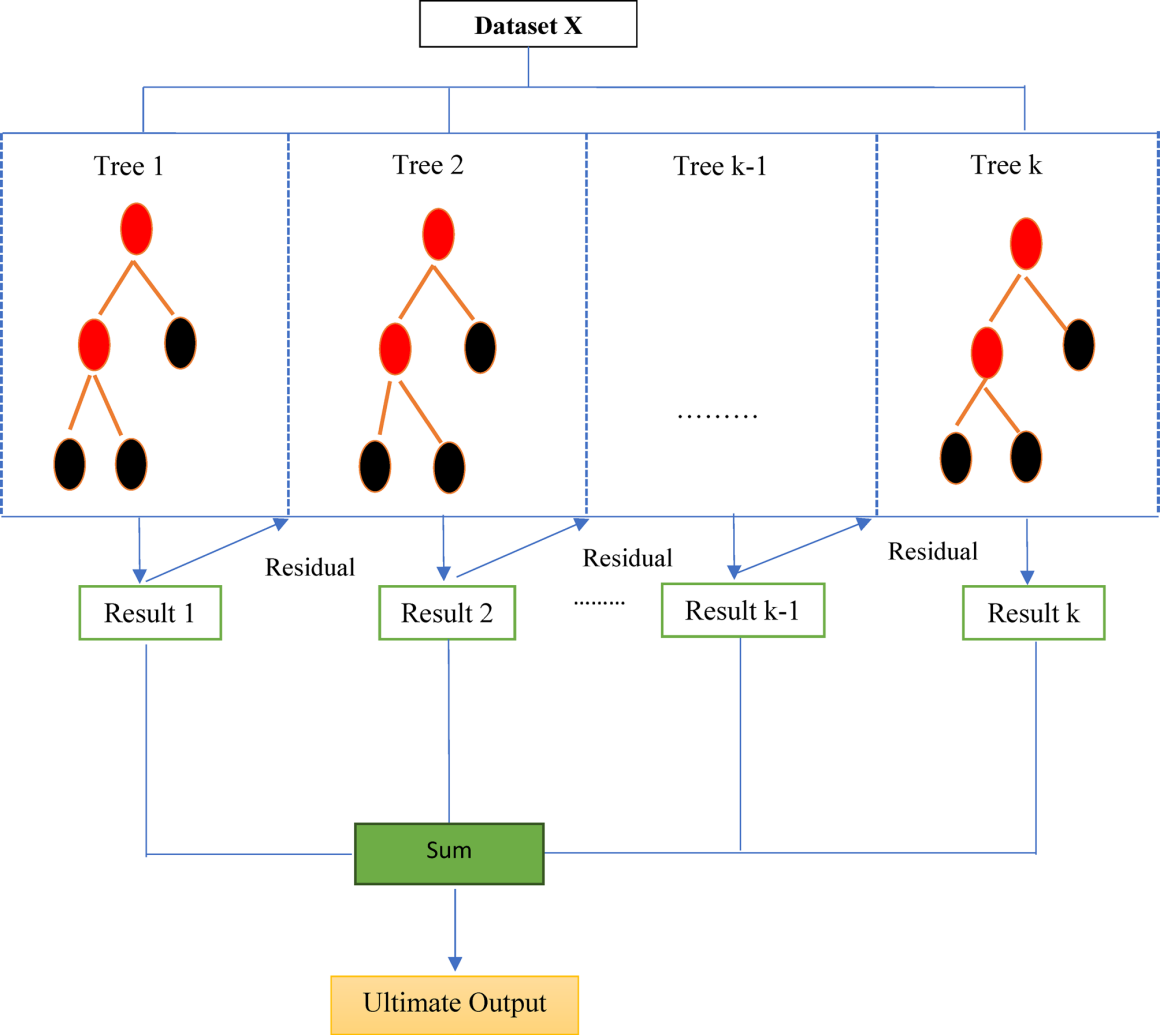
Schematic diagram of XGBoost model.

In the XGBoost framework (Fig. 3), the model starts with a base prediction, usually the mean of the target variable and computes the difference between observed and predicted values, called residuals. It then creates another decision tree that forecasts the residuals so that the new tree learns patterns in the errors and making subsequent predictions closer to actual values. The process stops after several iterations either when the residuals stop decreasing or the combined predictions become progressively closer to actual values. The gradient descent technique optimizes a differentiable convex loss function, and uses two regularization parameters (L1 and L2) to avoid overfitting^36,37^. L1 regularization reduces weights of unimportant features, while L2 regularization smooths leaf weights of trees.

The model parameters can be estimated by minimizing an objective function composed of a loss function and a regularization term. Mathematically, the model can be represented as^37^:

15 $$L^{\left( k \right)} = \sum\limits_{i = 1}^{m} {l\left( {y_{i} ,\hat{y}_{i}^{{\left( {k - 1} \right)}} + f_{k} \left( {x_{i} } \right)} \right) + \Omega \left( {f_{k} } \right)}$$

Here,

- *l* represents the loss function, which measures how much the predicted values deviate from the actual data $y_{i}$.
- $f_{k} \left( {x_{i} } \right)$ denotes the model of the $k^{th}$ tree for the $i^{th}$ data point.
- *t* is the iteration step in the optimization process.
- $\Omega \left( {f_{k} } \right)$ represents the regularization term.

The regularization term, which penalizes the number of leaves in the tree and the size of the leaves, is defined mathematically as:

16 $$\Omega \left( f \right) = \gamma T + \frac{1}{2}\lambda \mathop \sum \limits_{j = 1}^{T} w_{j}^{2}$$

where: T is the number of leaves in the tree, *γ* and *λ* are the regularization parameters called penalty coefficients, $w_{j}$ indicates the weight (score) of the leaf *j*.

The dataset was chronologically divided into training (60%), validation (20%), and testing (20%) subsets. The maximum number of boosting iterations was set to 10,000. Early stopping with a patience of 200 boosting rounds was employed by monitoring the validation RMSE to determine the optimal number of boosting iterations, which was subsequently fixed for all model runs to ensure consistent model complexity across experiments. The XGBoost model was implemented using the XGBoost Python library with the following hyperparameters: objective = ”reg:squarederror”, learning rate (η) = 0.03, maximum tree depth = 6, subsample ratio = 0.8, column subsampling ratio = 0.8, L2 regularization parameter (λ) = 1.0, and a fixed random seed of 42 to ensure reproducibility of the model training. The predictive performance was evaluated using the Nash–Sutcliffe Efficiency (NSE), Root Mean Square Error (RMSE), Mean Absolute Error (MAE), and the coefficient of determination (R^2^). Depending on the experimental configuration, the predictor variables consisted of either the original meteorological variables or the augmented feature set generated by the calibrated GR4J model, as described in the following section.

### Hybrid GR4J-XGBoost model

Since time series data regarding streamflow could include different attributes such as seasonality, a non-Gaussian error or heteroskedasticity, the hydrological behavior of catchments may not easily be depicted by stand-alone models^38^. The approximation of the GR4J model used to capture extreme flow events or complex nonlinear relationship may not be adequate as it provides only specific physically interpretable predictions. To address this, the hybrid framework combines the process-based GR4J hydrological model with the data-driven XGBoost algorithm to improve streamflow prediction. In the first stage, the GR4J model was calibrated using the training dataset and subsequently used to simulate the catchment hydrological processes from precipitation and potential evapotranspiration. The GR4J simulation can be mathematically represented as:

17 $$Q_{{{\mathrm{GR4J}}}}^{t} = f_{GR4J} \left( {P_{t} ,E_{t} ,X_{1} ,X_{2} ,X_{3} ,X_{4} } \right).$$

where, $P_{t}$ indicates the daily precipitation observed at time step t. $E_{t}$ indicates the daily potential evapotranspiration observed at time step t. $X_{1}$ represents the catchment’s soil water storage capacity. $X_{2}$ governs the interaction between groundwater and surface water. $X_{3}$ controls the temporary storage and routing of runoff within the catchment. $X_{4}$ defines the timing of the catchment’s response.

Unlike residual-based hybrid approaches, the present study adopts a feature augmentation strategy, in which the outputs and internal state variables of the GR4J model are incorporated as additional inputs into the XGBoost model. Specifically, GR4J-derived variables including simulated discharge (${Q}_{GR4J}^{t}$), production store state (S), production store inflow (P_s_), net precipitation (P_n_), actual evapotranspiration (E_s_), percolation (Perc), and net evapotranspiration (E_n_) are used. The meteorological inputs and GR4J-derived variables were therefore jointly used to represent both external climatic forcing and the internal hydrological state of the catchment.

**Fig. 4:**
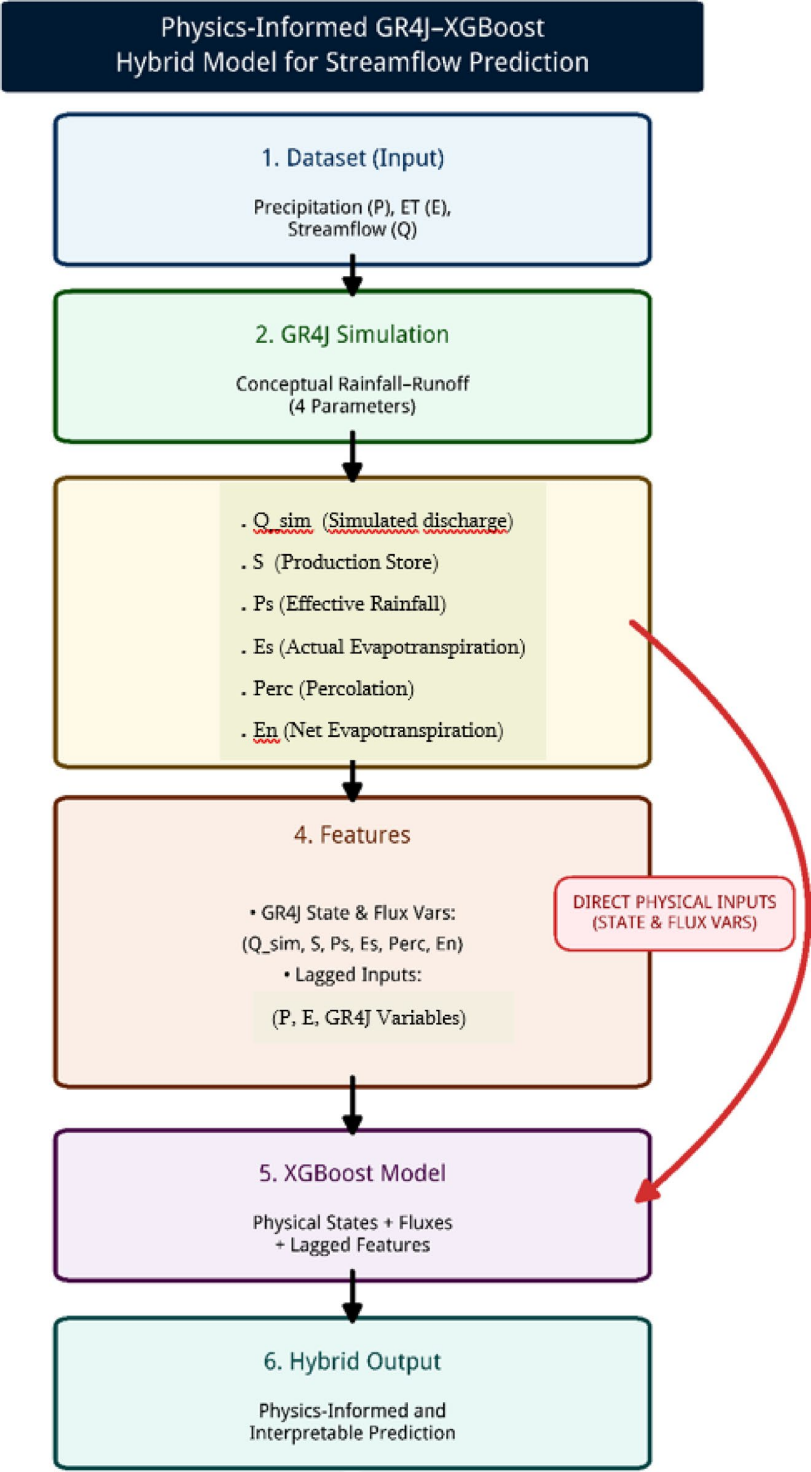
Framework of the proposed physics-informed GR4J–XGBoost hybrid model for streamflow prediction, incorporating GR4J-derived state and flux variables as inputs to the machine learning model.

To account for catchment memory, the current values and lagged values of all predictor variables were included. Lag configurations of 7, 14, and 30 days were evaluated independently. For a selected lag length L, the hybrid model can be expressed as:

18 $$Q^{t} = f_{XGBoost} \left( {Z_{t} ,Z_{t - 1} , \ldots Z_{t - L} } \right)$$

where

19 $$Z^{t} = \left[ {P_{t} ,E_{t} ,Q_{{{\mathrm{GR4J}}}}^{t} ,S^{t} ,P_{s}^{t} , P_{n}^{t} , E_{s}^{t} , Perc^{t} ,E_{n,}^{t} } \right]$$

denotes the combined meteorological and GR4J-derived predictor vector at time step t. The observed streamflow was used only as the prediction target and was not included among the lagged predictor variables. The overall architecture of the proposed hybrid framework is illustrated in Fig. 4.

This hybrid structure allows the machine learning model to exploit physically meaningful representations of catchment dynamics by integrating both GR4J-derived internal states and flux components. As a result, the model reduces its dependence on long lag structures and improves generalization across varying hydrological conditions. Furthermore, the inclusion of physically interpretable variables enhances model transparency and supports explainability analysis.

## Results

Using real in situ measured data including streamflow (*Q*), precipitation (P) and evapotranspiration (E), the performances of the GR4J, data-driven (XGBoost), and hybrid GR4J-XGBoost models were evaluated for streamflow prediction (Tables 1, 2, 3). To investigate the influence of catchment memory on model performance, the proposed models were evaluated using three lag configurations (7, 14, and 30 days), as presented in Tables 1, 2, 3. The models were compared based on NSE, RMSE, MAE, and R^2^ across training, validation, and test periods. All three models successfully reproduced the observed streamflow during the training period, although their predictive accuracy differed considerably. The RMSE, MAE, NSE, ranged from 0.033 to 3.656 m^3^/s, 0.024 to 2.234 m^3^/s, and 0.803 to 1.00, with average values of 1.468, 1.015, and 0.945, respectively (Table 1). However, clear differences in predictive accuracy were observed among the models.

**Table 1:** Performance comparison of conceptual (GR4J), data-driven (XGBoost), and hybrid GR4J–XGBoost models for streamflow prediction during the training period.

Model	Inputs		Training	
		RMSE, m^3^/s	MAE, m^3^/s	NSE
GR4J	P, E	3.656	2.213	0.803
XGBoost_7	P, E, (+ 7 lags)	3.042	2.234	0.864
XGBoost_14	P, E, (+ 14 lags)	1.171	0.875	0.980
XGBoost_30	P, E, (+ 30 lags)	0.033	0.024	1.000
GR4J-XGBoost_7	P, E + GR4J states (+ 7 lags)	0.986	0.727	0.986
GR4J-XGBoost_14	P, E + GR4J states (+ 14 lags)	0.804	0.599	0.990
GR4J-XGBoost_30	P, E + GR4J states (+ 30 lags)	0.584	0.435	0.995

GR4J states include En, Ps, Es, Perc, S, and Qsim. P: Precipitation, E: Reference (potential) evapotranspiration, En: Net evapotranspiration demand, Ps: Production store inflow, Es: Actual evaporation from production store, Perc: Percolation, S: Production store state (storage), Q_sim_: Simulated discharge. XGBoost-7, XGBoost-14, and XGBoost-30 denote data-driven XGBoost models using 7, 14, and 30 lagged inputs of P and E, respectively. GR4J-XGBoost-7, GR4J-XGBoost-14, and GR4J-XGBoost-30 represent hybrid models in which GR4J simulated states (En, Ps, Es, Perc, S, and Qsim) are combined with P and E, along with their corresponding lagged inputs.

**Table 2:** Performance comparison of conceptual (GR4J), data-driven (XGBoost), and hybrid GR4J–XGBoost models for streamflow prediction during the validation and test periods.

Model	Validation			Test		
	RMSE, m^3^/s	MAE, m^3^/s	NSE	RMSE, m^3^/s	MAE, m^3^/s	NSE
GR4J	4.230	2.576	0.803	4.206	2.487	0.678
XGBoost_7	5.678	4.227	0.428	6.217	4.089	0.297
XGBoost_14	4.917	3.669	0.569	5.165	3.417	0.514
XGBoost_30	4.850	3.420	0.584	4.671	2.945	0.604
GR4J-XGBoost_7	3.337	2.080	0.802	2.420	1.682	0.893
GR4J-XGBoost_14	3.309	2.080	0.805	2.631	1.774	0.874
GR4J-XGBoost_30	3.383	2.092	0.797	2.628	1.792	0.875

XGBoost-7, XGBoost-14, and XGBoost-30 denote data-driven XGBoost models using 7, 14, and 30 lagged inputs of P and E, respectively. GR4J-XGBoost-7, GR4J-XGBoost-14, and GR4J-XGBoost-30 represent hybrid models in which GR4J simulated states (En, Ps, Es, Perc, S, and Qsim) are combined with P and E, along with their corresponding lagged inputs.

**Table 3:** Ablation analysis comparing the reduced hybrid model (P, E, and Qsim) with the proposed hybrid model incorporating intermediate GR4J state and flux variables under the selected 7-day lag configuration.

	Training		Validation		Test	
Model	RMSE, m^3^/s	NSE	RMSE, m^3^/s	NSE	RMSE, m^3^/s	NSE
Reduced Hybrid (P,E,Qsim)	0.985	0.986	3.294	0.807	2.416	0.893
Proposed Hybrid	0.986	0.986	3.337	0.802	2.420	0.893

Both models were trained using identical data partitions, hyperparameter settings, and training procedures. The only difference between the models was the inclusion of the intermediate GR4J state and flux variables in the proposed hybrid model.

For the training dataset (Table 1), the hybrid GR4J-XGBoost model consistently outperformed the standalone GR4J model and achieved the best overall balance between prediction accuracy and model complexity for the 7- and 14-days lag configurations. For the 30 days lag configuration, XGBoost_30 achieved the best numerical performance during the training stage across all evaluation metrics. The GR4J model exhibited the lowest performance, showing a clear reduction in prediction accuracy compared to the other models. Compared with XGBoost_7, the GR4J-XGBoost_7 model reduced the RMSE to 0.986 m^3^/s and the MAE to 0.727 m^3^/s, while increasing the NSE to 0.986. The performance of the GR4J-XGBoost_7 model improved by 68% (RMSE), 67% (MAE), and 14% (NSE) relative to the XGBoost_7 model.

For the 14-day lag configuration, similar trends were observed. The GR4J-XGBoost_14 model achieved lower RMSE and MAE values than the XGBoost_14 model, while both models exhibited very similar NSE values. Specifically, the RMSE, MAE and NSE of the GR4J-XGBoost_14 model were improved by approximately 45%, 46% and 1%, respectively, compared to the XGBoost_14 model. This contrast highlights the importance of considering multiple performance metrics when evaluating and comparing predictive models.

For the 30-day lag configuration, the standalone XGBoost model achieved the best numerical performance during training. Under this configuration, the XGBoost_30 model achieves the best numerical performance among all evaluated models, with substantially lower RMSE and MAE values and an NSE value approaching 1.00. However, this advantage was not maintained during the validation and testing stages, suggesting that the additional lagged inputs mainly improved the fit to the training data rather than the model’s ability to generalize.

The lag length, extended up to 30 time steps, exerts a significant influence on model performance, and this impact varies between models XGBoost and GR4J-XGBoost. For model single XGBoost, increasing the number of lag inputs from 7 to 14 and up to 30 leads to a consistent improvement in performance. This enhancement may be attributed to the model’s ability to capture extended hydrological memory, thereby better inferring catchment storage effects and delayed hydrological responses. Substantial reductions in RMSE and MAE were observed as the lag length increased from 7 to 30. In contrast, the hybrid GR4J-XGBoost model exhibited relatively stable performance across different lag configurations. Although slight improvements were observed when increasing the lag length from 7 to 14 and 30, the differences remained comparatively small during the validation and testing stages. This stability suggests that the GR4J-derived hydrological states already capture a substantial portion of the basin memory and delayed runoff response, thereby reducing the dependence on extended lag structures and improving model robustness.

**Fig. 5:**
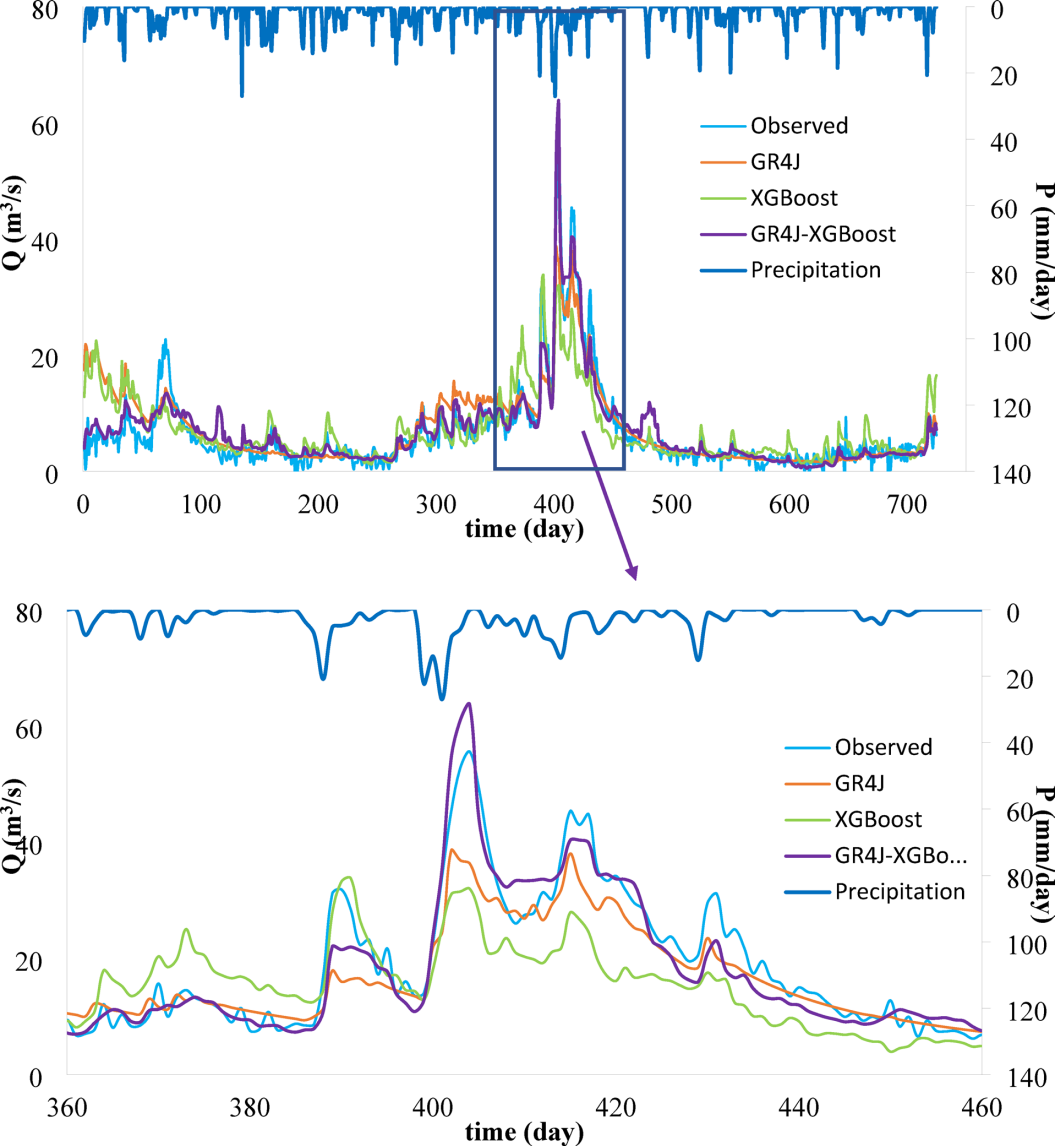
Time variation of observed and predicted streamflow: comparison of conceptual (GR4J), data-driven (XGBoost), and hybrid GR4J–XGBoost models for prediction during the testing period.

**Fig. 6:**
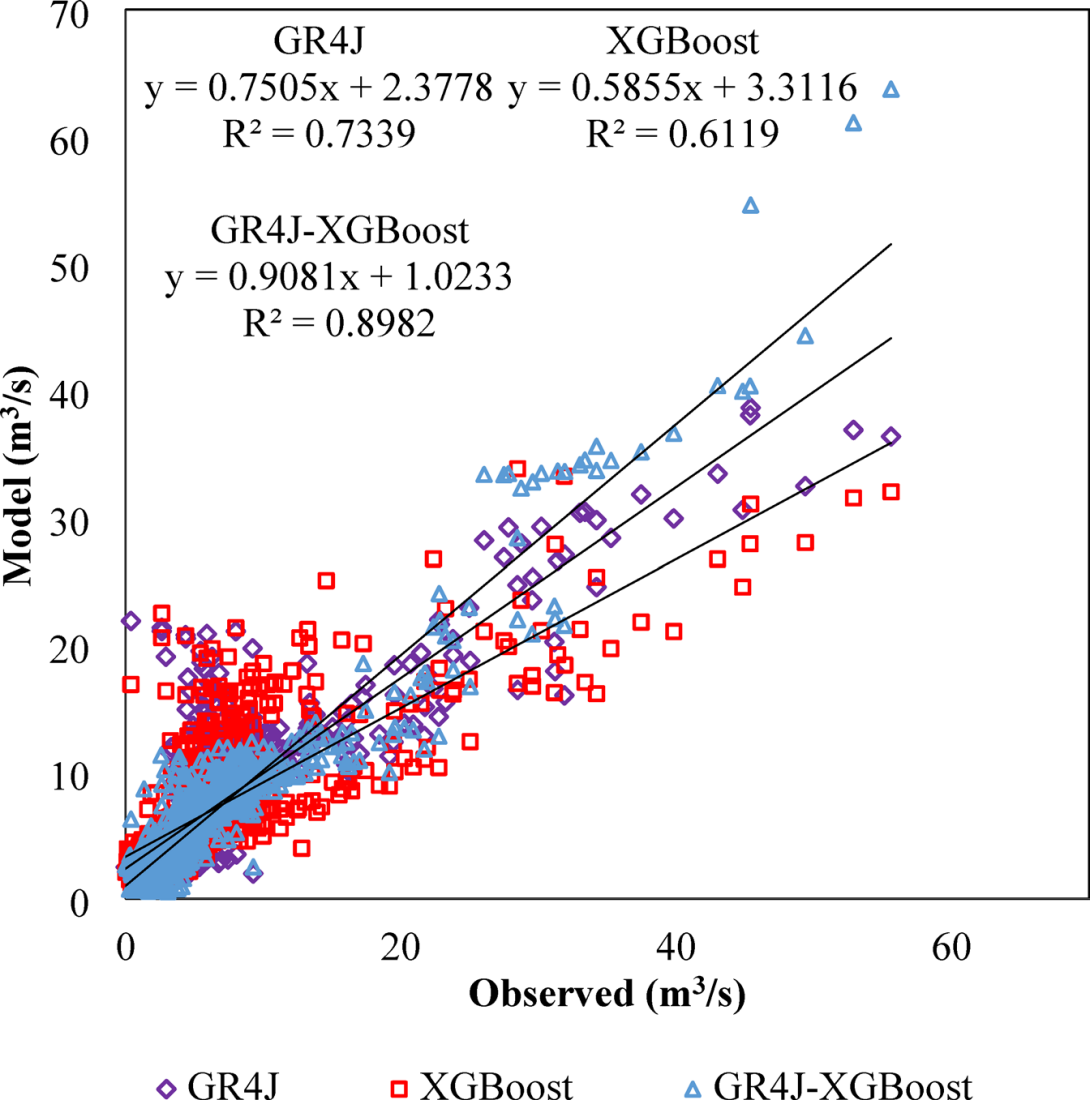
Comparison of conceptual (GR4J), data-driven (XGBoost), and hybrid GR4J–XGBoost models for prediction during the testing period.

Moving to the validation and testing stages (Table 2), it is observed that the numerical performance, measured by RMSE, MAE, and NSE, was somewhat lower for the validation and test subsets, as expected, with the notable exception of the GR4J model, for which the performance during the test stage was significantly improved. Overall, the hybrid GR4J-XGBoost model showed minimal degradation in performance across the training, validation, and test subset, although the performances on the test subset are slightly better than that observed on the validation subset. These results suggest that the GR4J-XGBoost model exhibits stronger generalization capability and is less prone to overfitting than the standalone XGBoost model. As shown in Fig. 5, the comparison between the observed and simulated values obtained by the GR4J, XGBoost, and the hybrid GR4J-XGBoost models indicates that the hybrid GR4J-XGBoost model provides the best agreement with the observed streamflow. In particular, it demonstrates a superior ability to reproduce extreme low and high flow conditions. Furthermore, the comparison based on scatterplots (Fig. 6), shows that the hybrid GR4J-XGBoost model exhibits less dispersion, indicating a better agreement between observed and predicted values. In contrast, the XGBoost model shows higher dispersion, reflecting lower prediction accuracy. However, the degradation in performance of the XGBoost model, whether at 7, 14, or 30 lags, is more pronounced, further highlighting the susceptibility to overfitting compared to the hybrid approach.

**Fig. 7:**
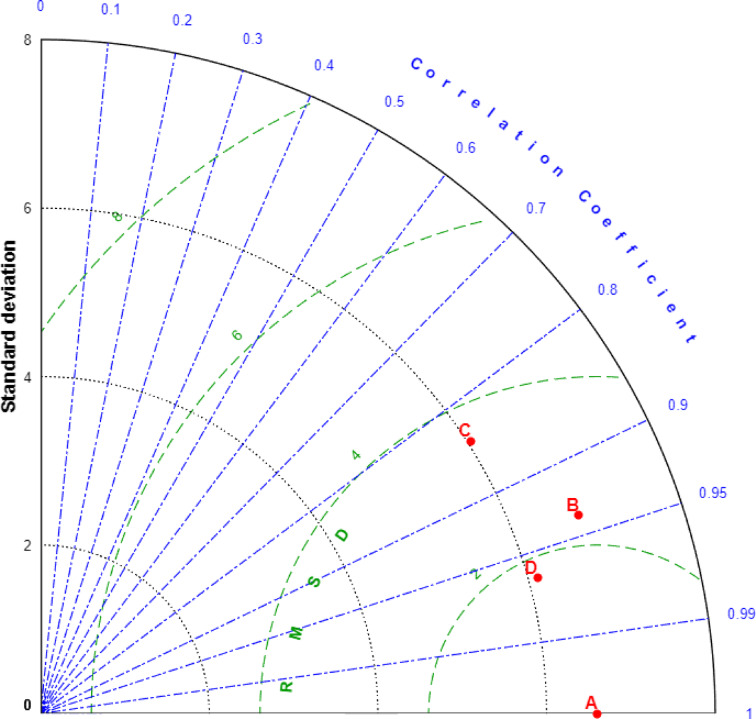
Taylor diagrams of the streamflow prediction models during the testing period. In the figure, (**A**): Observed streamflow, (**B**): GR4J, (**C**): XGBoost, and (**D**): GR4J–XGBoost.

**Fig. 8:**
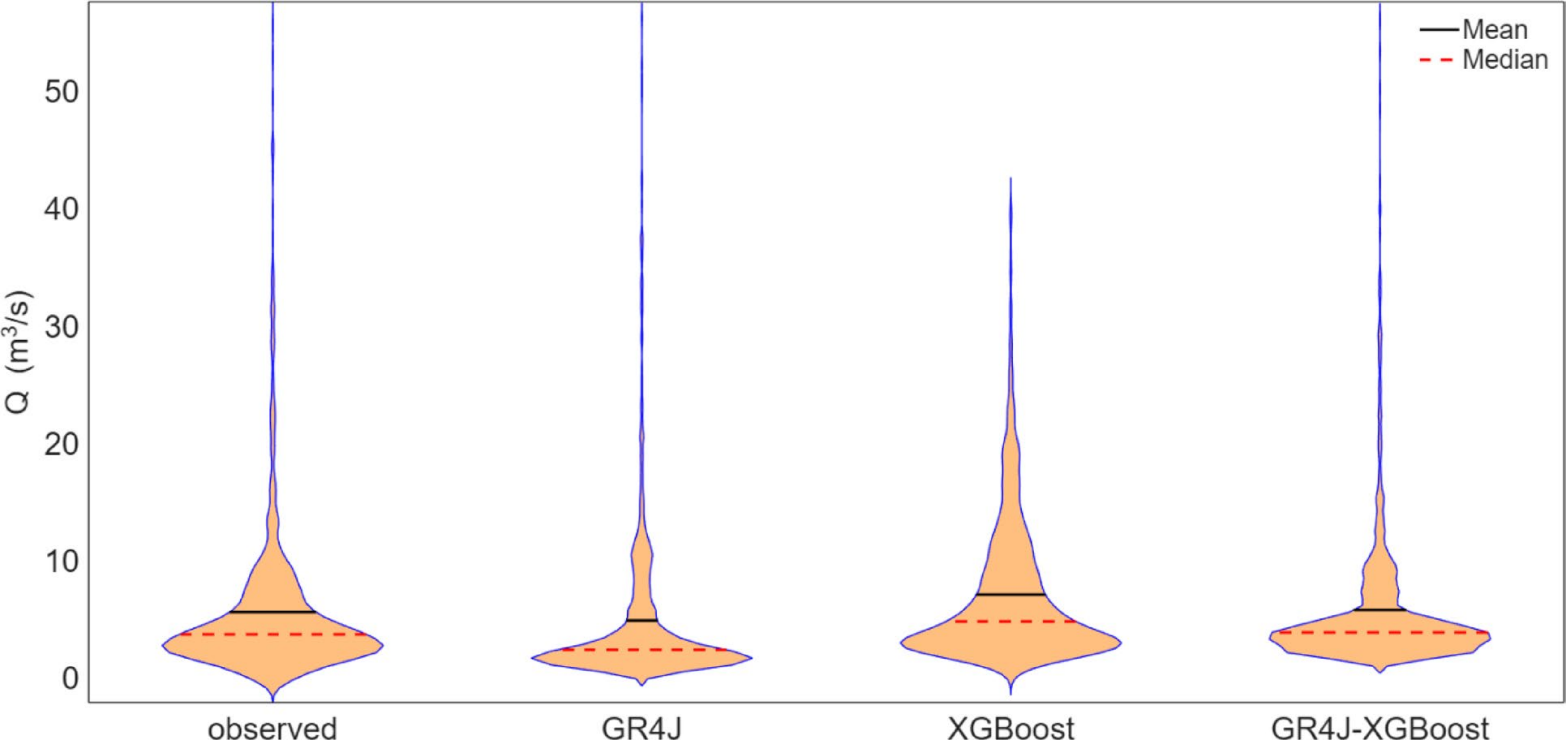
Violin plots of the streamflow prediction models during the testing period.

**Fig. 9:**
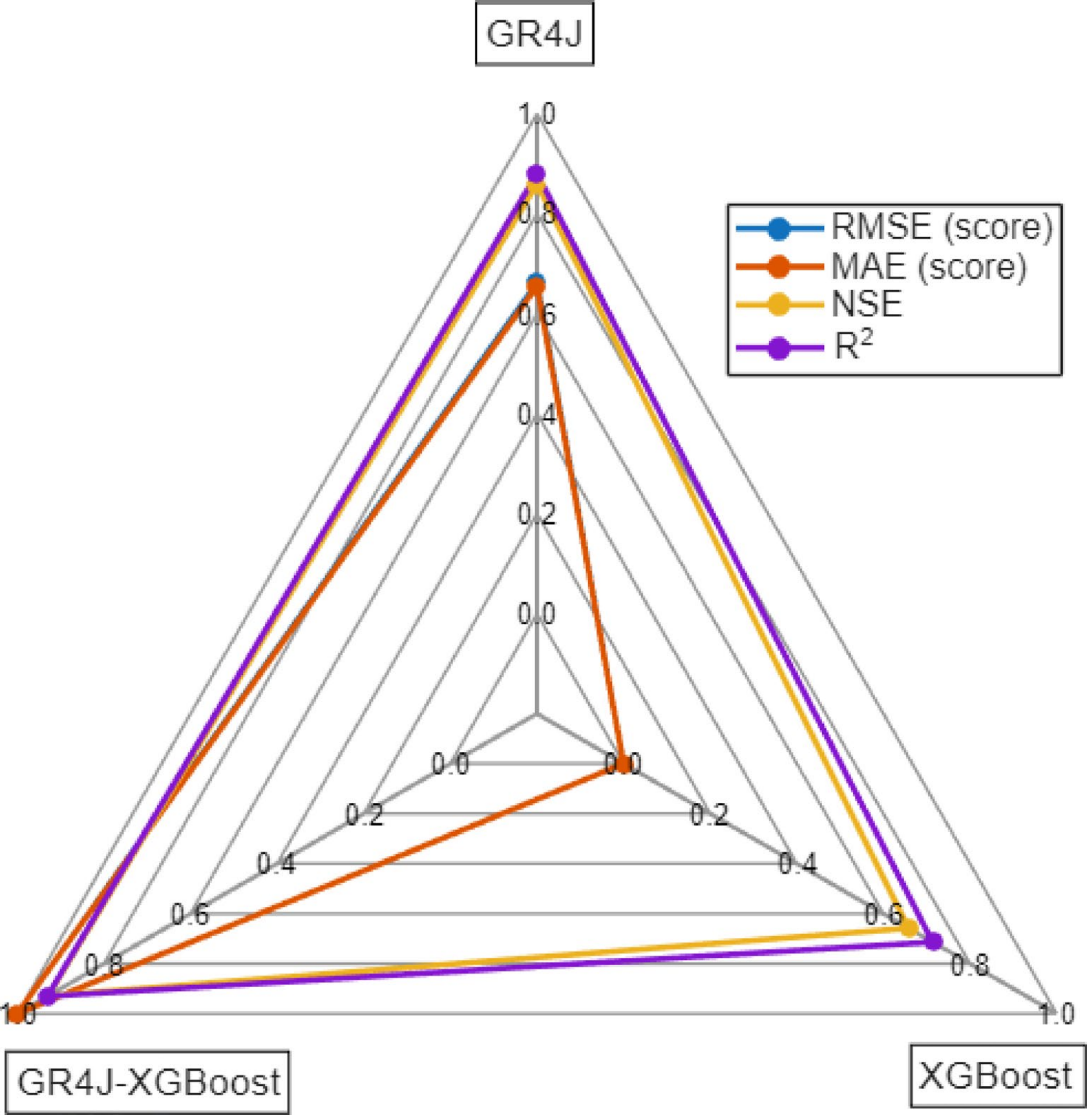
Spider plots of the runoff estimation models during the testing period. For visualization purposes, RMSE and MAE were converted into normalized performance scores (0–1), where higher values indicate better model performance.

By analyzing the validation results as depicted in Table 2, it can be concluded that the GR4J-XGBoost_7 model is markedly superior and more robust than XGBoost_7 model with improvements of 41.23% and 50.79% in terms of RMSE and MAE. Similarly, the GR4J-XGBoost_14 model improved the RMSE and MAE of the XGBoost_14 model by 32.70% and 43.31%, respectively. Finally, the GR4J-XGBoost_30 model improved the RMSE and MAE of the XGBoost_30 model by 30.25% and 38.83%. Although the performance of the hybrid GR4J-XGBoost model remains relatively stable across different lag configurations, a noticeable fluctuation is observed in the performance of model XGBoost. In particular, a clear improvement is recorded when increasing the lag from 7 to 30. During the testing stage, the standalone XGBoost models experienced a considerable degradation in predictive performance compared to the training stage, indicating reduced generalization capability and a tendency toward overfitting. In contrast, the hybrid GR4J-XGBoost models maintained consistently high predictive accuracy and robustness across all lag configurations. Specifically, the RMSE ranged from 2.420 to 2.631 m^3^/s, MAE from 1.682 to 1.792 m^3^/s, and NSE from 0.874 to 0.893, indicating only minor variations among the lag scenarios (Table 2). Moreover, the relatively stable performance across different lag configurations suggests that, beyond a lag length of approximately 7 days, the hybrid GR4J-XGBoost model was no longer able to extract substantial additional temporal information, as the hydrological memory is already effectively captured through GR4J-derived variables. The Taylor diagrams, violin plots, and spider plots (Figs. 7, 8, 9) further support the superiority of the hybrid model compared with the other evaluated models.

To further evaluate the contribution of the intermediate GR4J state and flux variables, an ablation analysis was performed using the selected 7-day lag configuration (Table 3). The reduced hybrid model (P, E, and Qsim) and the proposed hybrid model exhibited comparable predictive performance during both the validation and testing stages. Although the inclusion of the intermediate GR4J variables did not result in a substantial improvement in prediction accuracy, the proposed model maintained similar generalization performance while providing additional physically meaningful hydrological information for model interpretation.

**Fig. 10:**
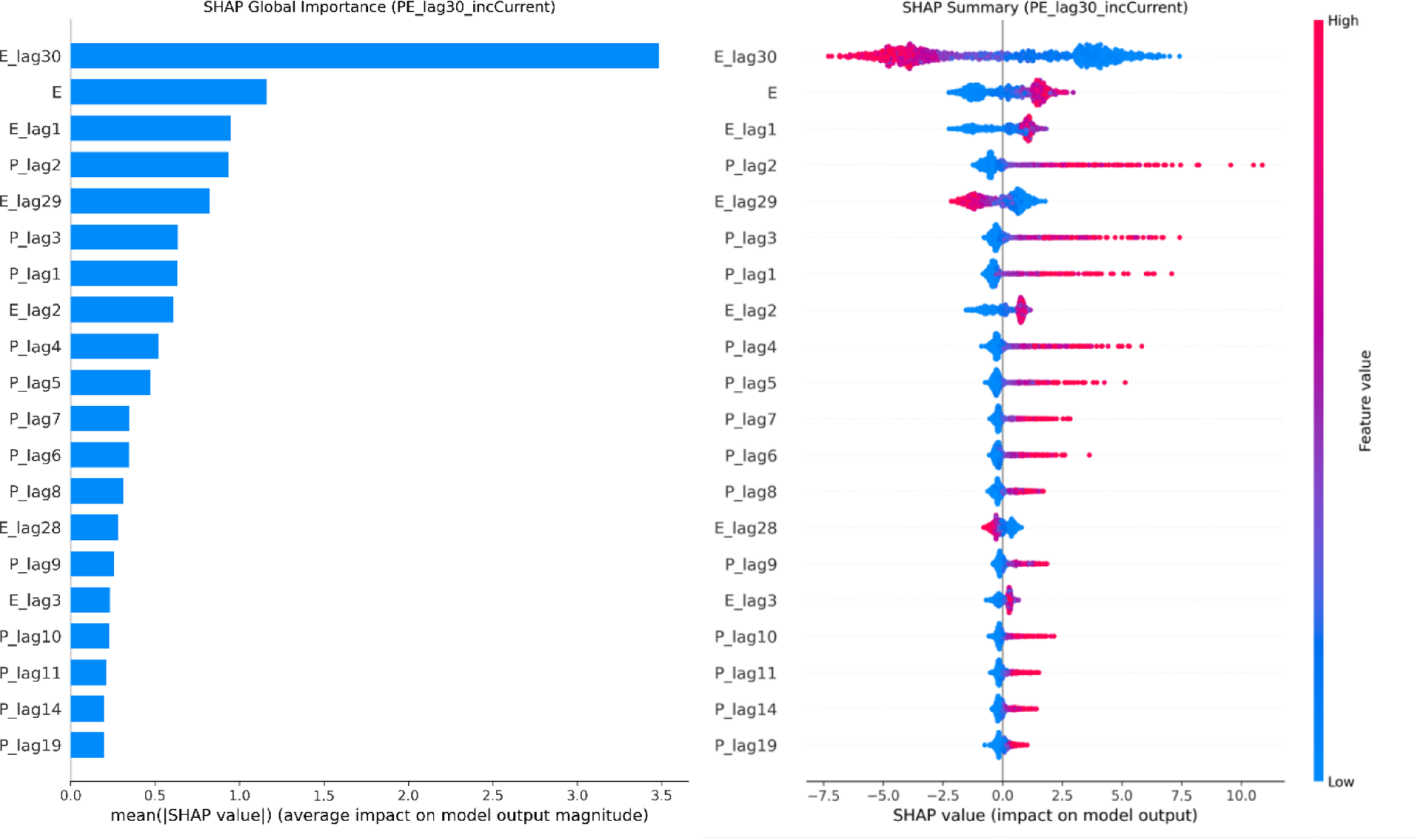
Global interpretability of the standalone XGBoost model based on meteorological inputs using SHAP analysis. The left panel presents the feature importance ranked by the mean absolute SHAP values, while the right panel shows the SHAP beeswarm plot illustrating the distribution and direction of feature impacts on streamflow predictions. The results indicate that the model predominantly relies on lagged precipitation (P) and reference evapotranspiration (E), reflecting a purely data-driven learning behavior in the absence of conceptual hydrological states.

Beyond numerical models’ comparison and prediction accuracy evaluation, Shapley Additive Explanations (SHAP) were used in the present study as a post-hoc interpretability tool. More importantly, SHAP was used to quantify feature importance by estimating the marginal contribution of each input variable, whether dominant or less influential. Both global and local interpretability can be done using SHAP, however, in the present study, only global interpretability was considered. Results obtained using the SHAP analysis for the XGBoost are depicted in Fig. 10 with two plots: the summary plot in the right panel and the bar plot in the left panel. The bar plot provides a list of features ranked by their calculated mean absolute SHAP values, while the summary plot denotes the SHAP value both positive or negative, indicating simultaneously the direction, i.e., positive or negative, and the magnitude of feature influence on the streamflow. In the summary plot each dot corresponds to one data, while the amplitude of the color helped in the identification of high or low values.

According to Fig. 10, the global analysis provided by the SHAP algorithm in the left panel given by its mean absolute Shapley value and taking into account all data samples, unveil a significant and consistently observed finding: the evapotranspiration E_lag30 dominates the XGBoost with a substantial difference compared to the other features (mean (|SHAP value|)≈3.5), followed by the E (mean (|SHAP value|)≈1.25), the E_lag1 and P_lag1 equally important with (mean (|SHAP value|)≈1.00), while the lowest contribution was registered with the lagged precipitation, i.e., lags 10, 11, 14, and 19. These results related to the stand-alone XGBoost model offer quantitative support that the streamflow is significantly governed by evapotranspiration history and current state, while the contribution of precipitation is more pronounced for the shorter lags, and precipitation’s contribution decreases as the lag horizon increases. The high relevance of evaporation with a lag of 30 days indicates that prior evaporation influences the state, functioning and finally runoff generation of the study basin. This long memory effect can be captured by the XGBoost model. However, the low contribution of some features like lagged precipitation, i.e., lags 10, 11, 14, and 19, which appear at the bottom of the ranking, does not reduce their underlying physical relevance, rather, we can highlight their marginal contribution compared to other hydrological factors.

**Fig. 11:**
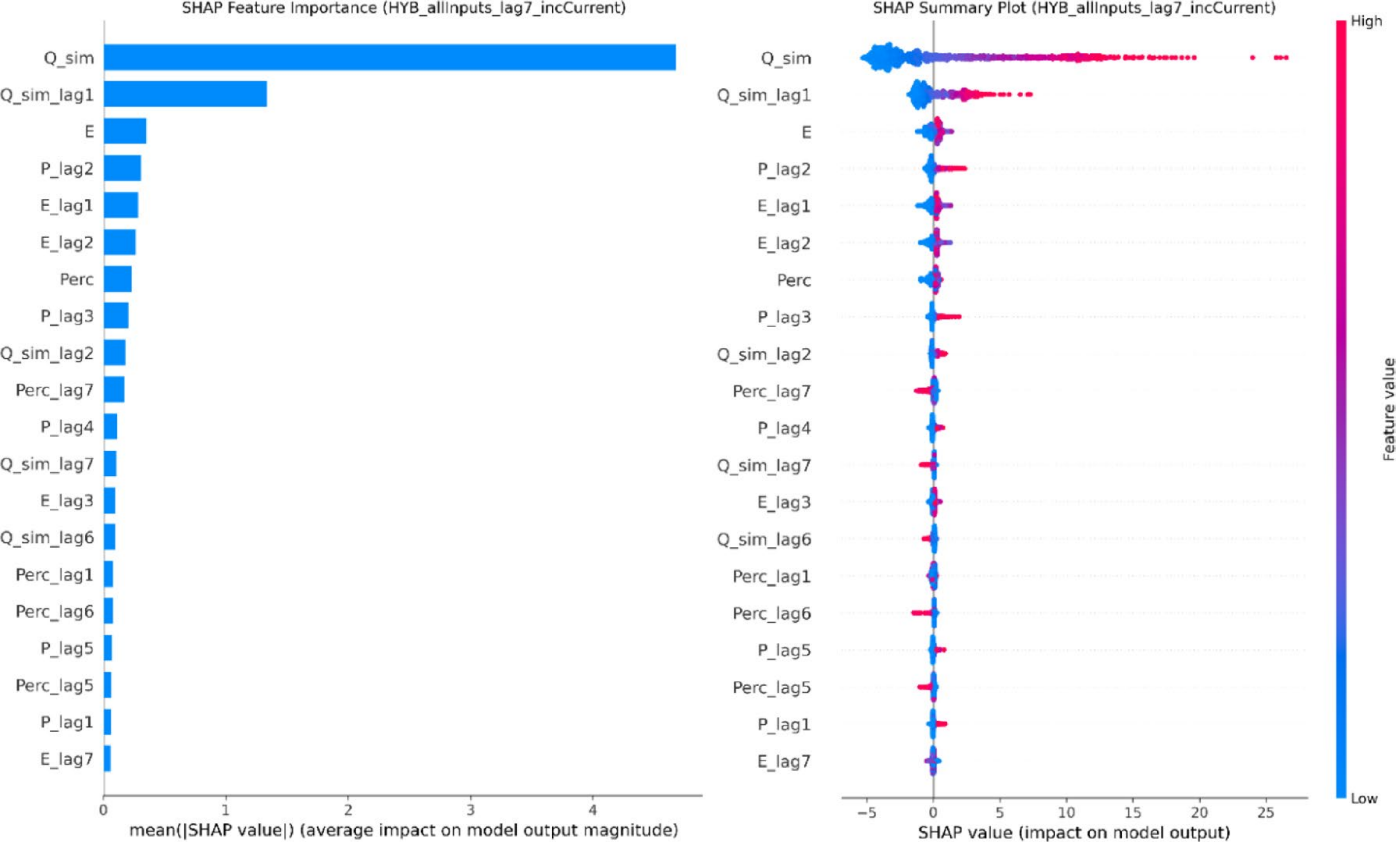
Global interpretability of the hybrid GR4J–XGBoost model using SHAP analysis. The left panel shows feature importance based on the mean absolute SHAP values, while the right panel presents the SHAP beeswarm plot illustrating the distribution and direction of feature impacts on streamflow predictions. Lagged GR4J state variables, particularly simulated discharge (Q_sim) and its temporal lags, dominate the feature rankings, highlighting the effective integration of conceptual hydrological states and data-driven learning in the hybrid framework.

Results obtained using the SHAP analysis for the GR4J-XGBoost are depicted in Fig. 11. SHAP analysis revealed that GR4J-derived state variables, particularly simulated discharge (Q_sim), production store storage (S), and percolation (Perc), were among the most influential predictors in the hybrid model. Lagged meteorological variables contributed to performance but were generally less dominant than physically meaningful state variables. According to Fig. 11, among all mean absolute SHAP values on bar plot (left panel), two features, Q_sim and Q_sim_lag1, are ranked very high compared to the other features having respectively a mean (|SHAP value|) of approximately ≈4.8 and ≈1.5, which can be considered as the key features for streamflow, and this indicates that, the hybrid model is largely governed by these features. The evapotranspiration (E) is ranked as the third influencing feature having a mean (|SHAP value|) of approximately ≈0.35 slightly superior to the values obtained by the P_lag1, E_lag_1, and E_lag_2. This observation complements the ablation analysis, indicating that although these variables did not substantially increase predictive accuracy, they contributed meaningfully to the internal decision-making process of the hybrid model.

**Fig. 12:**
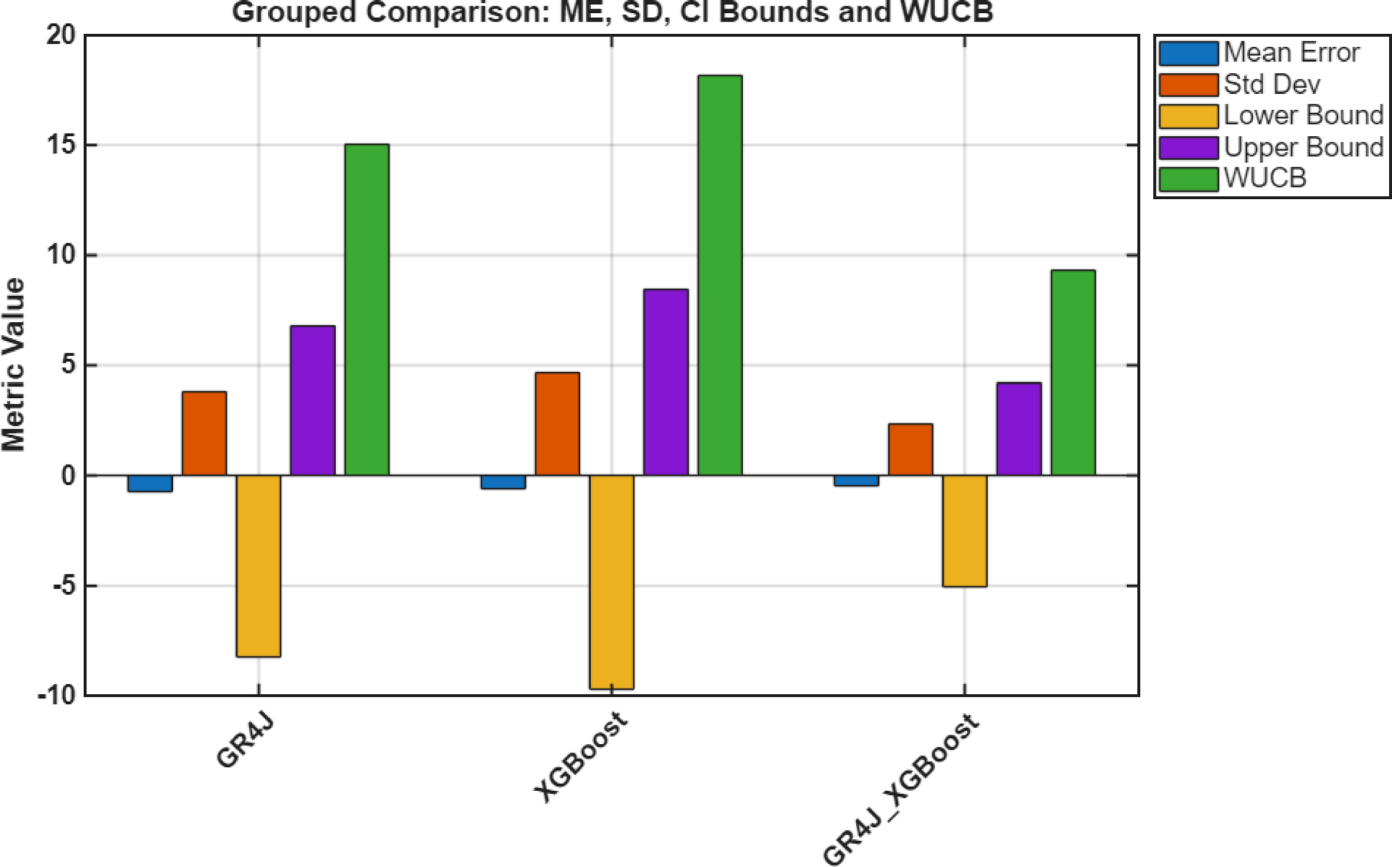
Bar charts for models’ uncertainty analysis.

The results of the uncertainty analysis are given in Table 4 and depicted in Fig. 12, which presents five uncertainty indicators: Mean Error (ME), Standard Deviation (Std Dev), lower and upper prediction bounds (LB and UB), and the Weighted Upper Confidence Bound (WUCB). The three models (GR4J, XGBoost, and GR4J-XGBoost) were compared based on these uncertainty metrics. Overall, the hybrid GR4J-XGBoost model exhibited the most reliable and stable predictive behavior, characterized by lower bias, smaller dispersion, and narrower uncertainty intervals compared to the standalone XGBoost and GR4J models. In contrast, the standalone XGBoost model showed considerably wider uncertainty bounds and higher variability, indicating reduced predictive robustness and stronger sensitivity to overfitting. The conceptual GR4J model demonstrated intermediate uncertainty characteristics, with moderate predictive stability but lower overall accuracy compared to the hybrid framework. Overall, the uncertainty analysis further confirms the superiority of the hybrid GR4J-XGBoost model in terms of uncertainty reduction, predictive reliability, and generalization capability.

**Table 4:** Summary of uncertainty metrics for streamflow prediction.

Model	Mean error	Std. dev	Lower bound	Upper bound	WUCB	Estimation bias
GR4J	− 0.7503	3.8346	− 8.266	6.7654	15.0314	Overestimation
XGBoost_30	− 0.6082	4.6349	− 9.6925	8.476	18.1685	Overestimation
GR4J-XGBoost_7	− 0.4237	2.3716	− 5.0718	4.2245	9.2964	Overestimation

Lower and upper bounds represent the 95% prediction interval limits. WUCB indicates the relative width of the uncertainty band; lower values correspond to sharper and more reliable predictions.

## Discussion

Machine learning (ML) and deep learning (DL) models have been rigorously evaluated for potential integration with conceptual and hydrological process models to enhance the accuracy and reliability of streamflow prediction. In this context, various published papers have made great advancement in improving our understanding of how this hybridization between black box and conceptual models can help in better capturing the process underlying the rainfall runoff process. Beyond conventional applications of ML and DL, numerous published studies have attempted to clarify various unexplored and ambiguous research pathways, particularly concerning the potential of ML and DL models enhancement by harnessing on the structural and process-based knowledge embedded in conceptual models. DL, i.e., the LSTM and process based hydrological models have their potentials and limitations, exhibiting varying levels of physical interpretability^39^. The present study extends these efforts by integrating GR4J-derived hydrological state and flux variables into an XGBoost framework and evaluating whether physically meaningful features can reduce the reliance on long lag structures while improving prediction robustness.

More recently, hybrid deep learning architectures have continued to improve runoff prediction by combining feature decomposition, attention mechanisms, transformer-based learning, and optimization strategies^27,28^. These studies demonstrated substantial improvements in predictive accuracy by enhancing network architectures and feature extraction. However, their primary emphasis remains on developing increasingly sophisticated data-driven models, whereas comparatively less attention has been given to integrating physically meaningful conceptual hydrological variables, explainable machine learning, and uncertainty quantification within a unified modelling framework.

Kumanlioglu and Fistikoglu^40^ were among the first researchers to demonstrate that GR4J and ANN models can be successfully integrated to improve river discharge prediction in Turkey. By employing Genetic Algorithms (GA) as an optimization technique, they showed that incorporating lagged precipitation and temperature as input variables significantly enhanced model performance. The hybrid GR4J-ANN-GA model was found to substantially outperform both the standalone GR4J and the conventional ANN models. These findings highlight the effectiveness of hybrid approaches in improving predictive accuracy, particularly when temporal dependencies are explicitly considered through lagged inputs.

Guan et al.^41^ conducted a study in Central Europe to assess the feasibility and potential benefits of hybridizing the GR4J hydrological model with LSTM and other modeling approaches. Their findings clearly demonstrated that the hybrid GR4J-LSTM model (mean NSE ≈ 0.601; mean RMSE ≈1.86) outperformed both the standalone LSTM model (mean NSE ≈0.562; mean RMSE≈1.94) and the standalone GR4J model (mean NSE ≈ 0.559; mean RMSE ≈ 1.92), respectively. These results confirm that integrating deep learning with conceptual models can enhance predictive performance by better capturing nonlinear hydrological dynamics.

Althoff et al.^42^ have modified the models of Kumanlioglu and Fistikoglu^40^ by replacing the ANN model with a Cubist regression model. In their study, two hybrid configurations were proposed. The first hybrid model, GR4J-Hyb1, considers only the input variables from the current day for the machine learning component. In contrast, the second hybrid model, GR4J-Hyb2**,** incorporates not only the current-day inputs but also additional lagged inputs from the previous three days. This modification allowed the authors to evaluate the added value of short-term temporal memory in improving the predictive performance of the hybrid GR4J-based framework. Using data from three-gauge stations within the Brazilian Cerrado biome, they demonstrated that, GR4J-Hyb2 exhibited superior numerical performances with R^2^ equal of ≈0.78, ≈0.91 and ≈0.94, respectively. These findings emphasize the importance of incorporating temporal information in hybrid models, highlighting that lagged inputs can significantly enhance model performance.

In a recently published study, Necesito et al.^43^ addressed an important and timely issue concerning the ability of DL models, particularly the LSTM, in modelling the complex rainfall-runoff relationship more effectively than traditional conceptual models. While the authors acknowledged the superior flexibility of LSTM in capturing complex nonlinear dynamics, they also highlighted its limitations in providing accurate and robust predictions of some extreme events. Their results revealed that the classical GR4J model tended to underestimate streamflow, whereas the standalone LSTM model systematically overestimated peak discharges. In contrast, the hybrid GR4J-LSTM model emerged as a balanced and intermediate solution that capitalizes on the strengths of both approaches. This hybrid framework tested using data from four sub-catchments in Samar, Philippines, enabled more realistic and robust estimation of both peak and low flows, achieving NSE values ranging from ≈0.63 to ≈0.84 and very low MAPE values (≤ 0.03). These findings demonstrate that hybrid models can effectively balance the biases of conceptual and data-driven approaches, leading to improved stability and reliability, particularly under extreme hydrological conditions.

In the same context, Tian et al.^44^ have investigated rainfall-runoff simulation using deep learning models based on four recurrent neural networks (RNN): the Elman recurrent neural network (ELM), echo state network (ESN), nonlinear autoregressive exogenous inputs neural network (NARX), and the LSTM, along with their hybrid versions developed through integration with the GR4J conceptual model. Their study placed particular emphasis on analyzing the uncertainties associated with simulated streamflows. Using data from two hydrological stations located in the Xiangjiang River and the Qujiang River basins in China, the authors demonstrated that LSTM and NARX models were more effective in capturing time-series dynamics compared to other RNN variants. Furthermore, the hybrid models substantially improved the simulation of high, median, and low flows. More specifically, they significantly reduced the bias related to the underestimation of peak discharges in the Xiangjiang River basin. In addition, the hybrid configurations reduced uncertainty intervals by more than 50% for median and low flows, while simultaneously increasing the coverage ratios of observed discharges. These findings highlight that hybrid approaches not only improve predictive accuracy but also substantially enhance uncertainty quantification and model reliability.

Sezen and Partal^33^ used data from three sub-basins of the Konya Closed Basin, Turkey, for modelling rainfall runoff process. They studied the capabilities of the GR6J and its hybrid version, i.e., the wavelet-based genetic algorithm-artificial neural network GR6J-WGANN. They compared between two versions: GR6J-WGANN-1 and GR6J-WGANN-2. According to the obtained results, the GR6J-WGANN-1 and GR6J-WGANN-2 that included various lags times of input data, were significantly superior to both the GR6J and WGANN models in terms of predictive accuracy and overall simulation performance. For example, at Sarisu station, GR6J-WGANN-1 was found to be the most accurate model exhibiting and NSE MAE and RMSE values of 0.61, 0.06 mm/day and 0.15 mm/day, compared to the values of 0.29, 0.11 mm/day and 0.21 mm/day, obtained using the GR6J model, while the values obtained by the WGANN based on precipitation an evapotranspiration were respectively, 0.23, 0.12 mm/day and 0.22 mm/day. These findings demonstrate that incorporating additional information through preprocessing techniques and lag structures can significantly enhance model performance.

Kapoor et al.^26^ hybridized the GR4J with two DL models namely, the LSTM and the CNN models and compared their performances in simulating the rainfall runoff process using data collected from 223 catchments in Australia. From the obtained results, they demonstrated that the hybrid GR4J-CNN-*Q*_*in*_ that included the observed antecedent streamflow (*Q*(*t*–1)) significantly contributed to the improvement of the hybrid models exhibiting the best numerical performances with mean NSE value of 0.825 compared to the values of 0.756, 0.754, 0.708, 0.437, 0.590, and 0.584, obtained using the GR4J-LSTM, GR4J-CNN, GR4J-LSTM, CNN, LSTM, and GR4J, respectively. These findings highlight the importance of incorporating antecedent information, particularly lagged streamflow, in improving hybrid model performance. Unlike Kapoor et al.^26^, who relied on antecedent observed streamflow as an input, the present study demonstrates that comparable predictive robustness can be achieved using GR4J-derived physically meaningful variables without directly incorporating observed streamflow lags.

Based on the studies discussed above, it can be clearly stated that hybridizing black-box models, particularly neural networks and deep learning approaches, with conceptual hydrological models has proven to be highly effective across a wide range of applications and data conditions. Although previous studies have focused either on overall simulation capability or on the modeling of extreme events (high, median, and low flows), the reported results have consistently been encouraging. These studies collectively demonstrate that hybridization enhances both predictive stability and reliability, especially under complex hydrological dynamics. However, most existing hybrid approaches primarily rely on lagged inputs or black-box learning mechanisms, with limited use of physically meaningful internal variables derived from conceptual models.

In comparison, the hybrid model developed in the present study, namely GR4J-XGBoost, explicitly incorporates GR4J-derived state variables as input features, enabling the model to capture hydrological processes in a more physically consistent and interpretable manner. The results demonstrate that this approach maintains competitive predictive performance while reducing dependence on long lag structures and improving the physical interpretability of the hybrid framework. The ablation analysis showed that the inclusion of intermediate GR4J variables did not produce a substantial increase in prediction accuracy relative to the reduced hybrid model. However, SHAP analysis confirmed that physically meaningful variables, particularly simulated discharge (Q_sim), percolation (Perc), and production store storage (S), actively contributed to model predictions, indicating that their principal value lies in improving process representation and interpretability rather than uniformly increasing predictive accuracy. Despite using multiple input configurations (7, 14, and 30 days lags), the hybrid model exhibits stable performance across all scenarios, indicating that essential hydrological memory is effectively captured through GR4J-derived variables. While the relevance of different lag-structures depends on the basin scale, soil type, geomorphology, and aquifer structure, the GR4J-XGBoost model can represent these differences in scale and basin characteristics in the XGBoost algorithm. Furthermore, the integration of SHAP-based interpretability and uncertainty analysis provides additional insights into model behavior, highlighting the robustness and reliability of the proposed framework. Overall, the GR4J-XGBoost model emerges as a physically informed and interpretable hybrid approach, offering a strong and competitive alternative to existing hybrid modeling strategies.

## Conclusions

This study introduced a hybrid modeling framework that combines the GR4J conceptual rainfall-runoff model with the XGBoost machine learning algorithm for streamflow prediction. The proposed approach uses GR4J-derived state and flux variables as meaningful input features, allowing the model to integrate process-based understanding with data-driven learning. The results show that the hybrid GR4J-XGBoost model outperformed both standalone GR4J and XGBoost models, particularly in terms of generalization capability and predictive stability. Although the standalone XGBoost model improved with longer lag times, it also showed performance degradation during validation and testing, suggesting potential overfitting. In contrast, the hybrid model maintained stable performance across all datasets and lag configurations. The ablation analysis further demonstrated that the inclusion of intermediate GR4J variables maintained comparable predictive performance relative to the reduced hybrid model while improving the physical interpretability of the framework through physically meaningful conceptual variables.

In particular, the integration of GR4J-derived flux components (En, Ps, Es, and Perc), together with storage state (S) and simulated discharge (Q_sim), enables the model to explicitly represent internal hydrological processes. A key finding of this study is that the inclusion of GR4J-derived variables effectively captures hydrological memory, thereby reducing the reliance on extended lag structures. This reduces the need for long lag structures, leading to a more robust model and a more efficient representation of catchment dynamics. The SHAP analysis further confirmed that physically meaningful variables, particularly simulated discharge and GR4J-derived state and flux components, play a central role in governing model predictions.

Moreover, the uncertainty analysis showed that the hybrid model consistently exhibited lower mean error, smaller uncertainty bounds, lower variability, and improved predictive reliability than the standalone GR4J and XGBoost models. These results highlight the benefits of combining conceptual and machine learning models not only for improving predictive accuracy but also for enhancing interpretability and uncertainty quantification.

Overall, the proposed GR4J-XGBoost framework offers a physics-informed and interpretable hybrid modeling approach, providing a strong alternative for rainfall-runoff modeling. Future research may explore the application and validation of this framework across multiple catchments, including ungagged basins, with different climatic conditions, basin scales, and hydrological characteristics, as well as its integration with other conceptual models and advanced optimization methods.

## Data Availability

The data presented in this study are available upon request from the corresponding author (contact ozgur.kisi@th-luebeck.de).
